# Sequence space coverage, entropy of genomes and the potential to detect non-human DNA in human samples

**DOI:** 10.1186/1471-2164-9-509

**Published:** 2008-10-30

**Authors:** Zhandong Liu, Santosh S Venkatesh, Carlo C Maley

**Affiliations:** 1 Genomics and Computational Biology Graduate Group, School of Medicine, University of Pennsylvania, Philadelphia, PA 19104, USA; 2 Department of Electrical and Systems Engineering, University of Pennsylvania, Philadelphia, PA 19104, USA; 3 Systems Biology Division, Molecular and Cellular Oncogenesis Program, Wistar Institute, Philadelphia, PA 19104, USA

## Abstract

**Background:**

Genomes store information for building and maintaining organisms. Complete sequencing of many genomes provides the opportunity to study and compare global information properties of those genomes.

**Results:**

We have analyzed aspects of the information content of *Homo sapiens, Mus musculus, Drosophila melanogaster, Caenorhabditis elegans, Arabidopsis thaliana, Saccharomyces cerevisiae*, and *Escherichia coli *(K-12) genomes. Virtually all possible (> 98%) 12 bp oligomers appear in vertebrate genomes while < 2% of 19 bp oligomers are present. Other species showed different ranges of > 98% to < 2% of possible oligomers in *D. melanogaster *(12–17 bp), *C. elegans *(11–17 bp), *A. thaliana *(11–17 bp), *S. cerevisiae *(10–16 bp) and *E. coli *(9–15 bp). Frequencies of unique oligomers in the genomes follow similar patterns. We identified a set of 2.6 M 15-mers that are more than 1 nucleotide different from all 15-mers in the human genome and so could be used as probes to detect microbes in human samples. In a human sample, these probes would detect 100% of the 433 currently fully sequenced prokaryotes and 75% of the 3065 fully sequenced viruses. The human genome is significantly more compact in sequence space than a random genome. We identified the most frequent 5- to 20-mers in the human genome, which may prove useful as PCR primers. We also identified a bacterium, *Anaeromyxobacter dehalogenans*, which has an exceptionally low diversity of oligomers given the size of its genome and its GC content. The entropy of coding regions in the human genome is significantly higher than non-coding regions and chromosomes. However chromosomes 1, 2, 9, 12 and 14 have a relatively high proportion of coding DNA without high entropy, and chromosome 20 is the opposite with a low frequency of coding regions but relatively high entropy.

**Conclusion:**

Measures of the frequency of oligomers are useful for designing PCR assays and for identifying chromosomes and organisms with hidden structure that had not been previously recognized. This information may be used to detect novel microbes in human tissues.

## Background

The discovery of the structure of DNA [1] was a fundamental event in biology because it illuminated the mechanism by which information can be encoded, passed on to future generations and even constructed by natural selection. With the sequencing of the human and other genomes [2,3], we are now able to compare the information encoded in the genomes of a wide variety of organisms and study the mechanisms that change genomes over time, including sequence mutations, recombination, duplication and deletion [4,5]. A great deal of research has focused on the coding and regulatory regions of genomes and regularly uses informational content measurements to analyze the structure of loci in the genome [6-8]. In addition, recent interest has turned to analyzing regions that do not encode proteins [9].

Relatively little is known about the global informational properties of most genomes. The conditional entropy was measured for human chromosome 22 [10] along with the frequency distributions of 3- through 7-mers in chromosomes 21 and 22 [11]. Stanley et al. analyzed the distribution of 1- to 4-mers within a wide variety of organisms and found that some tend to cluster within genomes (usually in non-coding regions) and others tend to "repel" each other [12,13]. Entropy measures have been applied to yeast and *C. elegans *whole chromosomes [14]. Recently, McHardy et al. showed that the distribution of 5-mer and 6-mer oligonucleotides in a > 1 kb fragment of DNA is characteristic of an organism and can be used in metagenomic studies to classify and construct the potentially millions of genomes in an environmental sample [15]. We are only beginning to compare informational properties between human chromosomes and across species.

The set of all oligonucleotides (oligos) of length n defines an n-dimensional discrete space, S^*n *^where each point is a possible *n*-mer and each dimension has only four states (A, C, G or T). Here we have measured the proportion of sequence space covered by oligos of length 1 – 20 in *H. sapien, M. musculus, D. melanogaster, C. elegans, A. thaliana, S. cerevisiae*, and *E. coli k12*. We have also measured the frequency of unique *n*-mers in those species and the compactness of the human genome in *n*-mer space. We also have identified all 5- to 20-mers that appear in the human genome more than 30,000 times, as well as the set of 15-mers that do not appear in the human genome and are more than 1 nucleotide different from all 15-mers in the human genome. In addition, we have measured the information content of the human genome for different oligo lengths and compared the information content to the proportion of coding regions in each human chromosome. The results match empirical observations and give a global view of the informational properties across a wide variety of genomes. Finally, we profiled the 10-mer space coverage for a wide range of 433 microbial genomes and found that the extent of sequence space coverage is largely determined by genome size and GC content.

## Results

### Sequence space coverage

**Figure 1:**
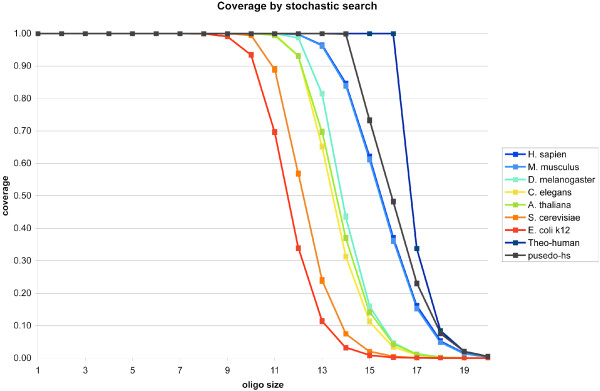
**The percentage of all possible *n*-mers (coverage) that appear in *H. sapien, M. musculus, D. melanogaster, C. elegans, A. thaliana, S. cerevisiae, E. coli k12*, theoretical and pseudo-human genomes.** Theo-human is the maximum coverage a human-length genome could achieve if every *n*-mer in its genome was unique. The pseudo-human (pseudo-hs) genome is a random genome generated with the same length and dinucleotide frequencies of the human genome. The space coverage of each genome listed above is plotted against the length of the oligomer analyzed, ranging from 1 to 20.

We randomly generated 5 sets of 100,000 probes for each oligo length n, and then determined the proportion of those *n*-mers present in the genomes of *Homo sapiens, Mus musculus, Drosophila melanogaster, Caenorhabditis elegans, Arabidopsis thaliana, Saccharomyces cerevisiae*, and *Escherichia coli k12*. The *n*-mer space coverage for each genome is plotted against oligo length n in Figure 1 (See additional file 1 for the data). The *E. coli *genome includes all 8-mers, and less than 0.21% of all 16-mers. In contrast, the human genome includes all 11-mers and less than 0.38% of all 20-mers. Of course, *n*-mer space coverage reflects genome size to some extent. For example, the human genome has a much higher coverage than the yeast genome for every oligo length. The 7 genomes that we investigated in this study differ the most in their coverage for 13-mer space, ranging from 11.4% coverage for *E. coli *to 96% coverage for human and mouse. For comparison, we also generated a random "pseudo" human genome with the same length and dinucleotide frequencies (see Appendix for a formal analysis of the expected number of *n*-mers in this pseudo-human genome). The fact that the true human genome has less coverage of *n*-mer space than the pseudo-human genome (Figure 1) shows that there are more repeated *n*-mers in the human genome than one would expect by chance.

**Figure 2:**
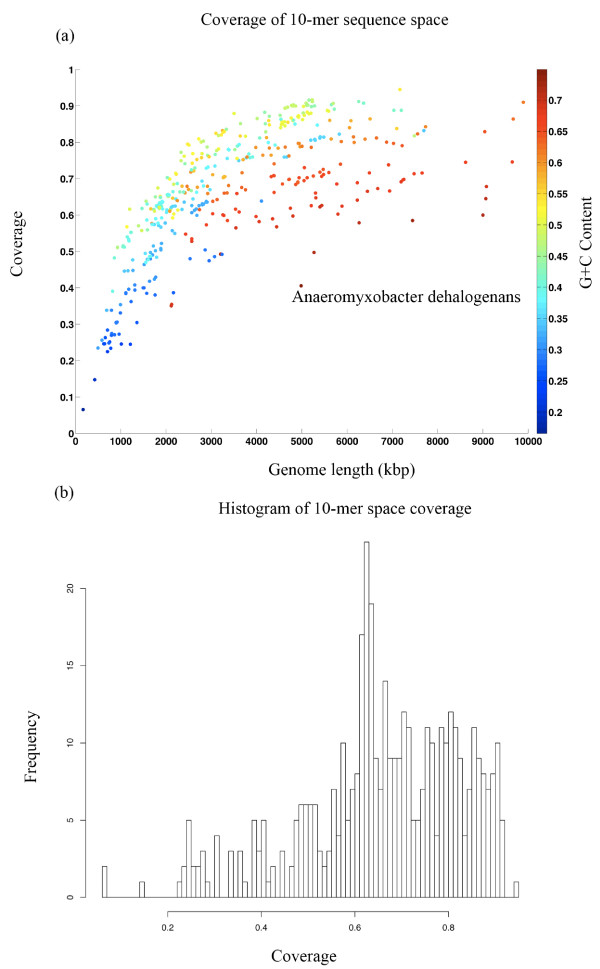
**(a) Coverage of 10-mer sequence space as a function of genome size in 433 fully sequenced microbial genomes.** The legend for the color-coding of GC content appears on the right. Smaller genomes have lower GC content. *Anaeromyxobacter dehalogenans *is an outlier with unusually low coverage for its genome size and GC content (outside of the 99.9% predicted interval). (b) A histogram for the proportion of the 10-mer sequence space covered by each of the 433 fully sequenced microbial genomes.

The coverage analysis of genomes can be used to analyze the complexity of any fully sequenced genome. We applied this analysis to 433 fully sequenced microbial genomes (Figure 2, see additional file 2 for the species, coverage, GC content and genome sizes). A multivariate regression of log genome size and deviation of GC content from 0.5 frequency, on 10-mer sequence space coverage, shows that variation in coverage can almost entirely be explained by genome size and GC content (adjusted R^2 ^= 0.92, p < 0.01). However, *Anaeromyxobacter dehalogenans *is an outlier with lower coverage than would be predicted by the model (actual coverage = 0.406, predicted coverage = 0.563, 99.9% predicted interval: 0.412–0.713). In addition, we confirmed the significant association between log genome size and GC content such that organisms with smaller genomes have lower GC content (Figure 2, linear regression p < 0.001) [16].

### Frequency of unique *n*-mers

**Figure 3:**
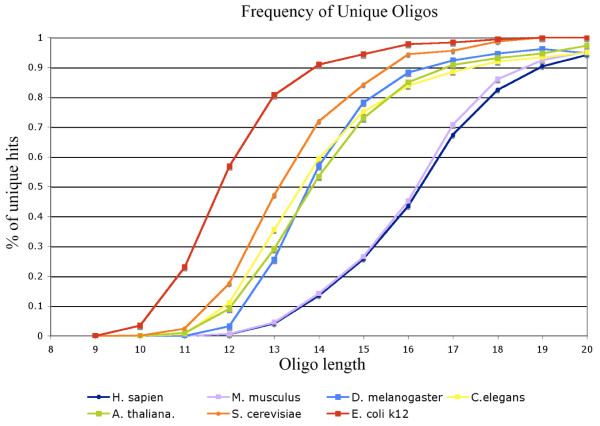
**The percentage of *n*-mers that appeared exactly once (unique hits), out of all the *n*-mers detected in each genome. **Slightly less than 50% of 16-mers detected in humans are unique. Whereas, for E. coli, a little more than 50% of 12-mers were unique.

To calculate the percentage of *n*-mers that appear only once in a genome, we implemented a program to estimate the unique hits among our stochastic search results (Figure 3). The human and mouse genome has only 5% unique hits among all the 13-mers they contain, while *Drosophila, Arabidopsis*, and yeast have only a single copy of more than 50% of their 13-mers. Figure 3 implies that, in order to have at least an 80% chance of hybridizing to a unique locus, a PCR primer for human and mouse genomes should be greater than 17 bp, while for *Drosophila, C. elegans*, and *Arabidopsis*, it should be greater than 15 bp.

### Accuracy of stochastic estimates

To assess the accuracy of our stochastic estimates for coverage in longer *n*-mers, we compared the stochastic results with exhaustive search results of all possible *n*-mers (Table 1). Due to the limitations of computational power, we could only carry out exhaustive searches up to 15-mer space. The 95% confidence interval of our stochastic sampling is small and it contains the true coverage. The confidence intervals also show that accuracy decreases as the dimension of our sampling space increases. However, it is clear from Table 1 that our 100,000 probes are statistically powerful enough to estimate the percentage of coverage for oligos up to length 20.

**Table 1:** Estimates and exhaustive calculations of the human genome coverage of *n*-mer Space

*n-mer*	*Mean Coverage from Stochastic Sampling*^*1*^	*95% Confidence Interval*		*Coverage from Exhaustive search*
12	99.726%	99.718%	99.733%	99.730%
13	96.416%	96.319%	96.514%	96.458%
14	84.470%	84.308%	84.633%	84.444%
15	62.041%	61.865%	62.217%	62.124%
16	37.065%	36.934%	37.197%	NA^2^
17	16.156%	16.058%	16.254%	NA
18	5.332%	5.278%	5.386%	NA
19	1.529%	1.508%	1.551%	NA
20	0.382%	0.369%	0.396%	NA

^1^100,000 *n*-mers were used to estimate coverage of the genome, repeated 5 times to estimate the 95% confidence interval.

^2^The set of all possible *n*-mers greater than 15 bp is too large to be exhaustively searched given our current computational resources.

### Detecting non-human organisms with non-human 15-mers

One potential use of genome coverage data is to use the *n*-mers that do not appear in the human genome as probes to detect microbes or genetic alterations in human samples. In order to do this, we must choose an oligo length long enough that there are some *n*-mers that do not appear in the human genome (i.e., > 13-mers), and long enough that some non-human *n*-mers are likely to appear in non-human organisms, yet short enough that that a large fraction of the non-human *n*-mers could be probed in a human sample (i.e., < 16-mers). We focused on 15-mers because 38% (406.7 M) of all possible 15-mers do not appear in the human genome. However, 404.1 M of these differ from the reference human genome by a single nucleotide and so may appear as single nucleotide polymorphisms (SNPs) in human samples. An oligonucleotide array of the remaining panel of 2.6 M 15-mers, that are at least 2 SNPs different from human sequences, could be constructed using current technology (e.g. Roche NimbleGen arrays). We simulated an oligonucleotide array hybridization experiment using these 2.6 M non-human 15-mers to determine the likelihood of detecting any of the fully sequenced microbes. This hypothetical array would detect 75% (2314 of 3065) viruses with a median of 3 positive non-human 15-mers (range: 0 to 1,705) and 100% (of 433) bacterial species with a median of 3,873 positive non-human 15-mers (range: 1 in the obligate endosymbiont *Candidatus Carsonella ruddii PV*, up to 92,127 in *Burkholderia 383*). Of course, some of the fully sequenced microbes are closely related to each other and so these estimates are not based on completely independent samples.

### Ultra-frequent *n*-mers

Some *n*-mers appear at extremely high frequency in the human genome. Among this set are some recognizable functional motifs. For example, the TATA box (5'-TATAAA-3') is ranked in the top 2% of all the possible 6-mers and the E-box (5'-CACCTG-3') is ranked in the top 11% of all possible 6-mers. We identified all the 5- to 20-mers that appear more than 30,000 times in the human genome. The top 20 ultra-frequent *n*-mers are shown in Table 2. A full list of high frequency *n*-mers is available in additional file 3.

**Table 2:** Ultra-frequent *n*-mers in the human genome

*5-mer*	*Freq*	*6-mer*	*Freq*	*7-mer*	*Freq*	*8-mer*	*Freq*
AAAAA	38,658,471	AAAAAA	19,638,479	AAAAAAA	12,559,969	AAAAAAAA	9,155,123
ATTTT	23,349,997	AAAAAT	9,299,025	AAATAAA	3,521,836	ATATATAT	1,863,212
TATTT	19,344,297	TATTTT	8,283,181	AAAAAAT	3,335,157	TGTGTGTG	1,701,426
AGAAA	18,271,461	AAATAA	7,271,302	AAAGAAA	3,255,464	TTTTAAAA	1,574,448
AAATT	16,119,174	AGAAAA	7,027,241	TTTTAAA	3,218,950	AAAATAAA	1,562,386
TTATT	15,608,707	TTTATT	7,015,408	TTATTTT	3,132,222	AAAGAAAA	1,467,003
TTTTC	15,579,017	TTTTAA	6,910,624	ATATATA	3,034,879	AAAAGAAA	1,444,255
CAAAA	15,364,161	TTTCTT	6,890,187	TTAAAAA	2,961,520	AAAAATTA	1,392,585
TTCTT	15,093,160	TTTAAA	6,820,966	TTTTCTT	2,913,461	TAAAAATA	1,363,753
TCTTT	15,053,533	AAAATT	6,559,595	TTTTATT	2,814,631	CCAGCCTG	1,308,899
CTTTT	14,643,705	TTCTTT	6,476,513	TAAAAAT	2,811,575	CAGCCTGG	1,266,975
CATTT	13,897,384	ATAAAA	6,284,494	AAAAATT	2,773,210	CTTTTTTT	1,238,232
AAACA	13,686,083	TTTTTG	5,979,892	TTCTTTT	2,693,599	AAAAAGAA	1,228,463
TTTGT	13,634,981	ATTTTA	5,829,423	AGAAAAA	2,614,232	CAAAAAAA	1,223,343
TAAAT	13,334,177	AAATAT	5,784,598	AAAATTA	2,572,747	AAAACAAA	1,216,247
ATATA	13,333,472	AAAAGA	5,708,574	ACAAAAA	2,554,220	TTTTTCTT	1,175,794
TGAAA	13,099,712	TTTTGT	5,622,008	TTTGTTT	2,514,354	ATTTATTT	1,171,982
ATATT	13,067,844	TATATA	5,551,066	TGTGTGT	2,500,827	TTTTGTTT	1,155,387
AAAAC	12,161,846	TGTTTT	5,550,449	TTTTTTA	2,460,695	TTAAAAAA	1,145,762
AGAGA	12,078,839	CTTTTT	5,515,339	TTTTTTG	2,436,880	AAAAAATA	1,143,587

*9-mer*	*Freq*	*10-mer*	*Freq*			*11-mer*	*Freq*

AAAAAAAAA	7,276,886	AAAAAAAAAA	5,952,617			AAAAAAAAAAA	4,945,619
TGTGTGTGT	1,424,846	TGTGTGTGTG	1,168,929			TGTGTGTGTGT	1,067,659
ATATATATA	1,253,711	ATATATATAT	967,302			CTGTAATCCCA	804,201
CCAGGCTGG	1,043,029	TGTAATCCCA	857,278			ATATATATATA	785,762
CTGGGATTA	921,482	GCTGGGATTA	840,776			TGTAATCCCAG	782,505
CCTGTAATC	917,990	GGATTACAGG	835,847			CCTGTAATCCC	762,833
GGATTACAG	916,302	CTGTAATCCC	826,256			GTAATCCCAGC	744,961
GAGGCTGAG	911,055	GGAGGCTGAG	810,203			CCTCAGCCTCC	729,303
GCTGGGATT	899,822	CTGGGATTAC	802,982			GAGGCTGAGGC	602,716
TGTAATCCC	897,744	CCTCAGCCTC	795,109			GCCTGTAATCC	579,838
GGAGGCTGA	891,406	AGGCTGAGGC	644,447			AAAATACAAAA	564,984
GTAATCCCA	887,639	CCAGCCTGGG	644,416			CCTCCCAAAGT	561,106
AGGCTGAGG	881,036	GCCTGTAATC	629,425			GGGAGGCTGAG	556,004
TTGGGAGGC	847,581	AAAAAAAAAG	626,192			AGGCTGAGGCA	555,758
CTTTTTTTT	794,742	TTTGTATTTT	619,817			CACTTTGGGAG	554,429
TTTTATTTT	780,751	TTTGGGAGGC	619,461			CTTTGGGAGGC	553,151
CAAAAAAAA	758,731	AAATACAAAA	611,794			AGTAGCTGGGA	546,426
AAAAAAAAT	741,719	CAAAAAAAAA	602,719			AAAAATACAAA	542,436
AAATACAAA	740,229	ACTTTGGGAG	600,711			CAGGCTGGAGT	536,630
CCCAGGCTG	740,090	TCAGCCTCCC	600,206			TCCCAAAGTGC	534,617

*12-mer*	*Freq*	*13-mer*	*Freq*			*14-mer*	*Freq*

AAAAAAAAAAAA	4,144,156	AAAAAAAAAAAAA	3,468,084			AAAAAAAAAAAAAA	2,889,704
TGTGTGTGTGTG	928,266	TGTGTGTGTGTGT	867,556			GTGTGTGTGTGTGT	775,928
TGGGATTACAGG	744,980	CTGTAATCCCAGC	687,709			CCTGTAATCCCAGC	639,010
CTGGGATTACAG	737,944	CTGGGATTACAGG	684,576			ATATATATATATAT	503,574
GCTGGGATTACA	727,608	ATATATATATATA	571,291			CTGGGATTACAGGC	478,945
ATATATATATAT	664,692	GCCTGTAATCCCA	520,611			AGCACTTTGGGAGG	459,948
GGAGGCTGAGGC	562,149	GCCTCCCAAAGTG	493,408			GCACTTTGGGAGGC	448,768
GCCTGTAATCCC	532,230	GGAGGCTGAGGCA	489,998			AAGTGCTGGGATTA	448,388
GCCTCCCAAAGT	527,552	CTCCCAAAGTGCT	486,025			AAAGTGCTGGGATT	445,915
TGCCTCAGCCTC	523,666	GCACTTTGGGAGG	474,289			CTCCCAAAGTGCTG	443,681
CCTCCCAAAGTG	522,504	CAGCACTTTGGGA	471,479			GGAGGCTGAGGCAG	437,319
TCCCAAAGTGCT	517,353	AGTGCTGGGATTA	468,425			CAAAGTGCTGGGAT	436,625
AAAAATACAAAA	513,200	TGCACTCCAGCCT	466,972			CAGGCTGGAGTGCA	436,504
GGGAGGCTGAGG	507,919	GAGGCTGAGGCAG	465,893			CCAGCACTTTGGGA	434,713
GCACTTTGGGAG	501,469	AAGTGCTGGGATT	464,827			TGCTGGGATTACAG	428,229
CCAGGCTGGAGT	499,800	ATCCCAGCACTTT	460,277			CCAGGCTGGAGTGC	425,904
AGGCTGAGGCAG	492,943	GCACTCCAGCCTG	454,751			CCTGCCTCAGCCTC	423,801
AGGCTGGAGTGC	486,794	TGTAATCCCAGCA	453,696			TCCCAGCACTTTGG	423,684
AGTGCTGGGATT	486,236	CCAGGCTGGAGTG	453,312			TGTAATCCCAGCAC	417,426
CAGGCTGGAGTG	484,927	TCCCAGCACTTTG	448,301			AGTGCTGGGATTAC	416,557

*15-mer*	*Freq*	*16-mer*	*Freq*			*17-mer*	*Freq*

AAAAAAAAAAAAAAA	2,397,399	AAAAAAAAAAAAAAAA	1,981,757			AAAAAAAAAAAAAAAAA	1,634,441
TGTGTGTGTGTGTGT	729,915	TGTGTGTGTGTGTGTG	662,157			TGTGTGTGTGTGTGTGT	624,344
GCCTGTAATCCCAGC	448,640	TAATCCCAGCACTTTG	408,698			TAATCCCAGCACTTTGG	386,886
ATATATATATATATA	446,009	ATATATATATATATAT	402,936			ATCCCAGCACTTTGGGA	385,642
AGCACTTTGGGAGGC	435,271	CCAAAGTGCTGGGATT	400,385			AATCCCAGCACTTTGGG	381,085
TAATCCCAGCACTTT	430,431	GCCTCCCAAAGTGCTG	398,210			AAAGTGCTGGGATTACA	375,216
CAAAGTGCTGGGATT	423,190	TCCCAGCACTTTGGGA	395,536			CTCCCAAAGTGCTGGGA	373,151
CCTCCCAAAGTGCTG	420,378	CCCAAAGTGCTGGGAT	392,938			AAGTGCTGGGATTACAG	369,056
ATCCCAGCACTTTGG	412,897	TGTAATCCCAGCACTT	390,400			GCCTCCCAAAGTGCTGG	368,350
CCAGCACTTTGGGAG	409,630	CCTCCCAAAGTGCTGG	388,489			CAAAGTGCTGGGATTAC	364,621
CCAGGCTGGAGTGCA	409,063	CTGTAATCCCAGCACT	385,038			CCCAGCACTTTGGGAGG	363,569
TGTAATCCCAGCACT	407,346	AAAGTGCTGGGATTAC	383,514			ATATATATATATATATA	363,206
CCCAGCACTTTGGGA	406,238	CCCAGCACTTTGGGAG	383,068			AGTGCTGGGATTACAGG	358,629
TCCCAGCACTTTGGG	403,080	GTGCTGGGATTACAGG	367,247			TCTACTAAAAATACAAA	340,685
AAGTGCTGGGATTAC	399,124	TGCACTCCAGCCTGGG	361,996			CTACTAAAAATACAAAA	340,602
CCTGCCTCAGCCTCC	398,260	TACTAAAAATACAAAA	360,995			CTCCTGCCTCAGCCTCC	328,239
TGCTGGGATTACAGG	398,070	GAGGCTGAGGCAGGAG	349,118			TTGTATTTTTAGTAGAG	325,500
GTGCTGGGATTACAG	394,430	CTACTAAAAATACAAA	348,511			TTCTCCTGCCTCAGCCT	324,096
GCACTCCAGCCTGGG	376,640	TCCTGCCTCAGCCTCC	346,669			TCTCTACTAAAAATACA	319,930
TTTTGTATTTTTAGT	374,747	TTGTATTTTTAGTAGA	346,193			GGCTGAGGCAGGAGAAT	318,655

*18-mer*	*Freq*	*19-mer*	*Freq*			*20-mer*	*Freq*

AAAAAAAAAAAAAAAAAA	1,345,821	AAAAAAAAAAAAAAAAAAA	1,104,496			AAAAAAAAAAAAAAAAAAAA	901,140
GTGTGTGTGTGTGTGTGT	569,504	TGTGTGTGTGTGTGTGTGT	536,547			TGTGTGTGTGTGTGTGTGTG	489,597
TCCCAAAGTGCTGGGATT	374,035	TAATCCCAGCACTTTGGGA	361,679			CTCCCAAAGTGCTGGGATTA	341,542
TAATCCCAGCACTTTGGG	368,428	AATCCCAGCACTTTGGGAG	353,065			AATCCCAGCACTTTGGGAGG	335,334
ATCCCAGCACTTTGGGAG	363,933	ATCCCAGCACTTTGGGAGG	345,608			GCCTCCCAAAGTGCTGGGAT	328,159
TGTAATCCCAGCACTTTG	356,809	TGTAATCCCAGCACTTTGG	338,285			GTAATCCCAGCACTTTGGGA	323,542
CTGTAATCCCAGCACTTT	354,805	CAAAGTGCTGGGATTACAG	337,559			CCCAAAGTGCTGGGATTACA	322,532
TCCCAGCACTTTGGGAGG	354,247	GCCTCCCAAAGTGCTGGGA	336,291			CTGTAATCCCAGCACTTTGG	320,216
GTAATCCCAGCACTTTGG	345,648	CCTGTAATCCCAGCACTTT	330,885			CAAAGTGCTGGGATTACAGG	315,005
CCCAGCACTTTGGGAGGC	345,007	CCCAAAGTGCTGGGATTAC	329,491			TTTTGTATTTTTAGTAGAGA	300,256
CCTGTAATCCCAGCACTT	343,923	CTCTACTAAAAATACAAAA	313,148			ATTCTCCTGCCTCAGCCTCC	280,115
TTTTGTATTTTTAGTAGA	333,004	TCTCTACTAAAAATACAAA	307,152			ATATATATATATATATATAT	279,576
ATATATATATATATATAT	332,710	ATATATATATATATATATA	302,986			TTTTTGTATTTTTAGTAGAG	272,798
TTTGTATTTTTAGTAGAG	320,328	GAGGCTGAGGCAGGAGAAT	296,297			CCACTGCACTCCAGCCTGGG	239,735
TCTCTACTAAAAATACAA	312,108	GGAGGCTGAGGCAGGAGAA	291,488			GCCTGTAATCCCAGCACTTT	238,538
AGGCTGAGGCAGGAGAAT	311,358	TTTTTGTATTTTTAGTAGA	290,073			GGCCTCCCAAAGTGCTGGGA	224,951
GAGGCTGAGGCAGGAGAA	308,372	CCAGGCTGGAGTGCAGTGG	273,189			GTCTCTACTAAAAATACAAA	195,075
TCTCCTGCCTCAGCCTCC	298,682	CACTGCACTCCAGCCTGGG	256,220			TTCTCCTGCCTCAGCCTCCC	191,155
TTTTTGTATTTTTAGTAG	296,585	GCCTGTAATCCCAGCACTT	247,562			GCCACTGCACTCCAGCCTGG	183,392
CCAGGCTGGAGTGCAGTG	292,150	GGCCTCCCAAAGTGCTGGG	230,427			TTTTTTTTTTTTTTTTGAGA	167,522

### Density in sequence space

**Figure 4:**
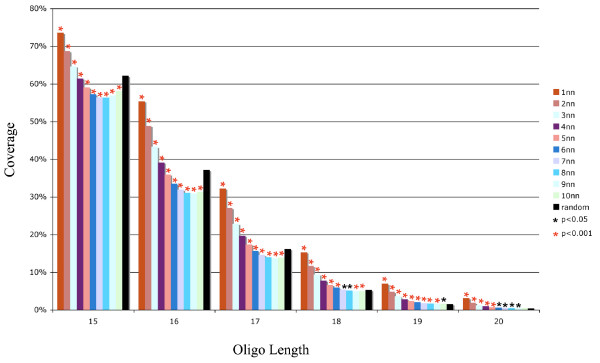
**The density of the human genome in sequence space.** For every randomly generated *n*-mer that was detected in the human genome, we generated all single basepair variants (3n variants for each *n*-mer) and tested them to see if they were also represented in the human genome (1nn). We also generated 3n of the 2 bp variants (2nn), 3n of the 3 bp variants, and so on up to variants that differed in 10 bp from the original human *n*-mer. The sequences that are only a few SNPs away from the original human *n*-mer are significantly more likely to be in the human genome compared to a random *n*-mer (black bars, "random"). This shows that the human genome is relatively compact in sequence space. The standard error for all points is < 0.003.

Based on our simulation, the pseudo-human genome has much higher space coverage than the true human genome for every oligo length (Figure 1). This leads to the hypothesis that human genomes are much more compact in sequence space than would be expected by chance. To explore this hypothesis, we examined how many 1 bp variants of human *n*-mers are also in the human genome. For each of the *n*-mers found in the human genome, we generated its 3n different 1 bp variants and then scanned the human genome for the presence of these variants. We found that significantly more 1 bp variants of human oligos were also in the human genome compared to random *n*-mers (Figure 4). The same is true for 2–4 bp variants of human oligos (Figure 4). Thus, the human genome is more compact, or dense, in sequence space than a random genome. When we generated variants that were more distant from the original human *n*-mer (up to 10 bp variants), we found in some cases that distant oligos were less likely to be in the human genome than a random oligo (Figure 4).

### Entropy rate

**Figure 5:**
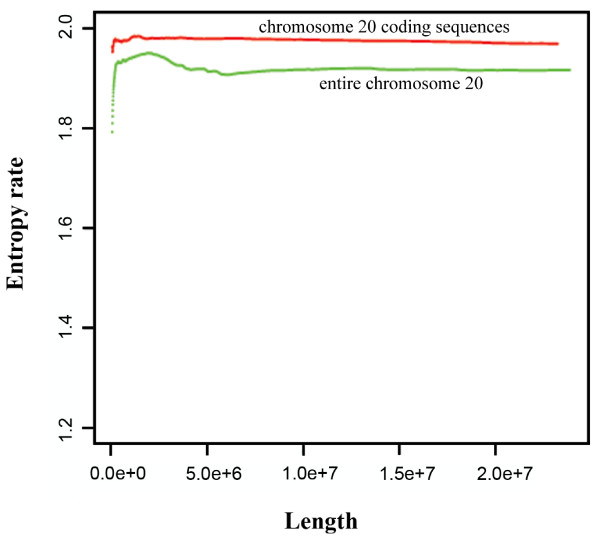
**Entropy rate, using the Lempel-Ziv 77 algorithm, for the coding sequence (red) and the genomic sequence for chromosome 20 (green), as a function of the length of the sequence analyzed. **The entropy calculation converges after 10 million bases.

**Figure 6:**
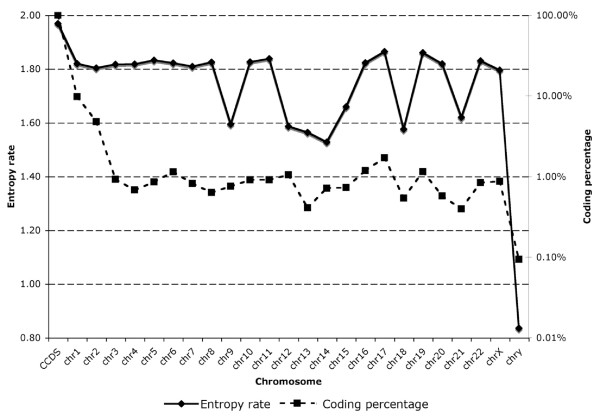
**The entropy, or information content (solid line, left Y axis) and percent of the sequence coding for proteins (dashed line, right Y axis, log scale) for each human chromosome as well as the full set of coding regions (CCDS). **Given the higher entropy rate of coding regions to non-coding regions, we expect a correlation between the two measurements. However, chromosomes 1, 2, 9, 12, and 14 have a lower information content than might be expected for the percent of those chromosomes occupied by protein coding regions. Chromosome 20 appears to have a higher entropy than would be expected given its gene poor content. This may be a signal of extensive non-protein coding, yet functional RNA on chromosome 20.

Previous studies have examined the entropy, or information content, within regions of a genome [6-8]. We calculated the information content of each human chromosome. This facilitates comparisons between chromosomes. We used the Lempel-Ziv 77 algorithm to estimate the entropy rate of both coding sequences and whole genomic sequences for the human genome [17]. Coding sequences have a higher entropy rate (information content) than genomic sequences (Figure 5). Note that the highly repetitive regions of telomeres and centromeres are generally coded as non-specific bases ("N") in the human genome and so are excluded from our calculations. Entropy was calculated from either the entire chromosome, for chromosomes < 130 Mb, or the first 130 Mb of longer chromosomes. This is sufficient to generate stable estimates of the entropy (Figure 5). Figure 6 shows both the entropy and the percent of the chromosome filled by coding regions for each human chromosome.

## Discussion

We have provided an overview and comparison of some of the informational properties of fully sequenced genomes. We found that virtually all oligomers of length less than 13 are represented in the human genome but only a vanishingly small proportion (< 1.53%) of oligos of length greater than 19. The mouse genome is the same in these respects. Similarly, very few oligos less than 13 bp are unique in the human genome, but the vast majority of oligos of length greater than 19, except repeat elements, are unique in the human genome. This is consistent with practical experience in the design of primers for PCR. Some of the most frequent *n*-mers in the human genome are microsatellites and ALU elements, as would be expected (Table 2). These ultra-frequent *n*-mers should be useful as high density markers in the genome and as primers for assays such as random amplified polymorphic DNA (RAPD) [18] in which a large number of regions of the genome may be amplified in a single PCR reaction. In fact, both microsatellites and ALU elements have been exploited for DNA fingerprinting [19-21]. The ultra-frequent *n*-mers we found are akin to the pyknons identified by Rigoutsos et al., except that pyknons need only appear 40+ times in the human genome, must be at least 16 bp long, and have the additional constraint that they appear in both protein coding and non-coding regions [22].

Whiteford et al. have analyzed a measure of the frequency of unique oligos in a variety of genomes [23]. However, their measure is subtly different from ours. The Whiteford measure of uniqueness essentially shatters a genome into *n*-mers and asks what proportion of those *n*-mers only occur once in the genome. This is appropriate for analyzing high throughput, short sequencing reads, since high copy-number *n*-mers will represent a large portion of the reads. Our measure asks what proportion of distinct *n*-mers only occur once in a genome? Thus, increasing the copy number of an *n*-mer already present in the genome would not change our statistic but would decrease the Whiteford measure of the frequency of unique *n*-mers. Our analysis suggests that the 25+bp reads of current high throughput sequencers are unlikely to produce sequences that would appear by chance in a genome other than the genome being sequenced (Figure 1). These longer *n*-mers should only be shared between species due to descent from a common ancestor.

The human genome is not spread evenly across sequence space but is rather compacted in closely related sequences (Figure 4). Compaction in sequence space may be the result of molecular evolution. Duplication events followed by divergence between the duplicated regions is thought to be a common mechanism for genome evolution [5] and would lead to such compaction. Similarly, transposons and other repeat elements lead to structure in the non-coding region of the genome that can be detected in the difference between the entropy of the coding regions versus the non-coding regions (Figure 5). Previous work has used Renyi entropy to address the problem of failure of convergence for entropy measures on short DNA sequences [24]. We have used the simpler and more traditional definition of entropy because convergence is not a problem for the analysis of whole human chromosomes (Figure 5).

The difference between the entropy of coding versus non-coding regions of the genome has long been known [14] and may help to explain why chromosomes 13, 18, 21 and the Y chromosome appear to have relatively low entropy compared to the rest of the genome (Figure 6). The correlation between nucleotides at varying distances ("mutual information") is also known to be higher in coding regions than non-coding regions [25]. However, there are a number of chromosomes for which the entropy does not track with the proportion of coding regions in chromosome. Chromosomes 1, 2, 9, 12, and 14 have relatively high proportions of coding regions without relatively high entropy while chromosome 20 has a relatively low proportion of coding regions without relatively low entropy (Figure 6). This may be a signal of functional, non-coding RNA on chromosome 20, for chromosome 20 does not have an unusually low frequency of repeats, an unusual G+C content, or an unusual density of CpG islands [2,3,26]. All of chromosome 20 is conserved as a single segment in the mouse chromosome 2 [2], suggesting it contains little junk DNA. However, the anomaly of non-protein coding information content on chromosome 20 cannot yet be explained by an over-abundance of miRNAs. Out of the 475 currently confirmed miRNAs in the human genome, 11 are located on chromosome 20 [27,28]. This is no more than would be expected by chance (Binomial probability of 11 or more miRNAs, p = 0.46).

Gaps remain in most of the sequenced genomes, but these are unlikely to significantly affect most of our analyses. In the sequence files, missing nucleotides are coded as N's and are skipped over by our algorithms. Build 35 of the human genome was missing 225 Mbp of the human sequence (7% of the genome), 200 Mbp of which is made up of heterochromatin [29]. Heterochromatin is highly repetitive sequence, including telomeres and centromeres. Since the sequenced part of the genome often extends past the borders of heterochromatin [29], it is likely that most of the *n*-mers in the heterochromatin (for n < 20) would have been counted in our analyses of coverage and uniqueness. The remaining 25 Mbp of euchromatic gaps are often associated with segmental duplications and copy number variations between subjects used for the reference sequencing [30]. Again, many of the *n*-mers in those gaps are probably represented elsewhere in the sequenced genome. However, the absence from our analysis of the 7% of the human genome with highly repetitive heterochromatin means that our estimates of entropy in the human genome (Figure 6) are probably slightly higher than the true values.

Coverage of sequence space is probably not subject to selection in and of itself, except in specialized cases of diversifying selection, such as occurs in the evolution of major histone compatibility complex (MHC) [31] and some testis genes [32]. However, coverage of sequence space may be a metric of evolvability because it represents the library of genetic sequences that may be duplicated, recombined and modified to generate new genes and functions. All things being equal (including genome size and mutation rates), we would predict that a population of organisms with greater coverage of sequence space should evolve more quickly to new environmental pressures than a population of organisms with fewer sub-sequences. This could be tested in evolvability experiments on bacteria with different degrees of sequence space coverage but similar mutation rates and genome sizes.

When we performed the analysis of coverage versus genome size in 10-mer sequence space. *Anaeromyxobacter dehalogenans *stands out as having an exceptionally low sequence coverage for its genome size and GC content (outside of the 99.9% predicted range). *A. dehalogenans *is an anaerobic bacterium with a GC content of 75%. It is able to reduce a variety of metals including ferric iron and Uranium (VI) and has been studied for its potential role in bioremediation [33,34].

One potential use of our results would be to develop assays to detect non-human organisms and sequences in human tissue samples. We found that with a panel of 2.6 M 15-mers that are at least 2 SNPs different from human 15-mers, we could easily detect all bacterial genomes and 75% of fully sequenced viruses. This approach is inspired by the negative selection algorithm used by the immune system: generate random amino acid sequence (peptide) detectors and then remove those that match self. Patterns of positive probes on an array of non-human 15-mers are likely to be enough to identify known microbes. To identify an unknown microbe, any non-human probe that hybridized to DNA from a human sample could be used as a PCR primer to sequence in both directions from that probe and thereby generate longer sequences of the non-human DNA. This would be important both for identifying pathogens in the etiology of diseases as well as for identifying symbiotic microbes that have received little attention because they either do not cause disease or they only cause disease through their absence. Such an array could also identify non-human sequences generated through insertions, deletions and translocations in cancer where such lesions may be targeted for therapy [35,36]. A number of other approaches have been taken to identifying non-human organisms in human samples. Cellular organisms can be identified by sequencing the 16S rRNA genes in the sample [37-40], though this misses viruses. DeRisi and colleagues have developed an oligonucleotide array with 70-mers of highly conserved sequences within most fully sequenced virus families [41]. This was used to identify the SARS virus as a coronavirus[42,43], though the array may not identify novel viruses that are dissimilar from the known viral families. A brute force metagenomics approach involves sequencing all the DNA or RNA in the sample and removing any sequences that match the human genome [44-47]. Currently, efforts are proceeding to sequence all the microbes found in the human body [48].

We have focused on an elegant and relatively simple metric of genome complexity: sequence space coverage. It can be calculated exhaustively or, more efficiently, through sampling based on a set of randomly generated oligos. While sequence space coverage is clearly influenced by genome size and GC content, we have also shown that the human genome is more compact in sequence space than a random genome. This is probably the signature of molecular evolution. Our measurements of sequence space coverage and entropy allow for the comparison between genomes and between chromosomes within a genome. This has led to the detection of outliers that may help to reveal properties of organisms and chromosomes that are not currently understood. Coverage data can also be used in a negative selection algorithm to develop assays to detect novel microbes in tissue samples.

## Methods

We used the Human Genome NCBI build 36 version 1, the reference Mouse sequence for C57BL/6J (NCBI build 36), and Escherichia coli strain K-12 substrain MG1655 (accession number U00096). See additional file 4 for all the build numbers and accession IDs of the genomes for Figure 1. All genomes sequences can be found on . The build number for all the bacteria genomes for Figure 2 can be found in our additional file 5. Genome statistics are based on all chromosomes and reverse complement strands for each organism.

### Exhaustive search

To find all the oligonucleotides of length n that are present in a genome is difficult for large n, due to computational memory constraints. We exhaustively searched the genomes for all possible 1- to 15-mers. The algorithm follows:

1. Given oligo length n, create a boolean array of size 4^n^

2. For each chromosome and its reverse complement:

a. For each nucleotide location *i *in the chromosome:

i. Convert the *n*-mer at location *i *into an array *index *(coding it as a 2n bit number with each nucleotide represented by 2 bits).

ii. Set the Boolean array [*index*] = true

3. Divide the number array locations set to true by the size of the array (4^n^).

### Stochastic sampling

The set of all oligonucleotides of length n has cardinality of 4^n^. The size of sequence space increases exponentially with respect to the oligo length. For example, if n = 18, the set contains 8G different oligos. For large n, it is impossible to save all the entries in the space to the memory of any 64-bit machine. Therefore, we choose to use stochastic sampling to estimate the sequence space coverage of the genomes as follows:

1. Randomly generate 100,000 *n*-mers and save them into a hash table

2. For each *n*-mer in the hash table:

a. For each chromosome and its reverse complement:

i. Scan the chromosome and record the number of times the *n*-mer appears in the genome

3. Calculate the coverage by dividing the total number of *n*-mers that appeared at least once in the genome by 100,000.

We found the stochastic sampling algorithm accurately estimates the true coverage of the genomes (Table 1).

### Identification of Ultra-Frequent Oligomers

In order to identify all oligomers of length 10–20 bp that appear > 30,000 times in the human genome we:

1. Identified all 10-mers that appeared in the human genome > 30,000 times by exhaustive search

2. Let *K *= the number of *n*-mers previously identified as appearing > 30,000 times. We generated 4*K *(*n*+1)-mers by concatenating one of the four possible nucleotides (A, C, G or T) to the end of the *K n*-mers that appeared > 30,000 times in the human genome.

3. We counted the number of times the 4*K *(*n*+1)-mers appear in the human genome and discarded any that appear less than 30,000 times.

4. If *n*+1 < 20, we incremented n and looped to step 2 with the new list.

This algorithm is guaranteed to find all *n*-mers that appear at least 30,000 times in the human genome. If a 20-mer occurs > 30,000 times in the human genome, then all prefixes of that 20-mer must occur at least as many times as the 20-mer. So the 10-mer prefix must also occur > 30,000 times and so would have been identified in step 1. The 11- to 19-mer prefixes, as well as the final 20-mer, would then have been generated in step 2 and not discarded in step 3. Therefore, all 20-mers that appear > 30,000 times are identified by the algorithm. This algorithm can be extended to arbitrary length oligomers for any positive lower bound on the frequency of the oligomers. We chose this approach, rather than the brute force approach of counting the frequency of all *n*-mers in the human genome because the above algorithm need only maintain a small list of the ultra-frequent *n*-mers at any step and so is more computationally efficient.

### Lempel-Ziv estimators of entropy rate

The Lempel-Ziv algorithm [49] is a computationally efficient scheme for universal data compression. The algorithm requires no *a priori *knowledge of source statistics (hence is "universal"), is particularly elegant, has a very low computational complexity, and produces very compact descriptions of large alphabets. These virtues have established the algorithm firmly as the standard data compression algorithm for the transmission and storage of large files over the Internet and on computers.

Given a sequence of letters from an alphabet, the algorithm parses the string sequentially to produce a dictionary of new phrases, in order of occurrence in the sequence. Specialized to the genome, the algorithm proceeds as follows.

The four phrases corresponding to the single nucleotides 'A', 'C', 'G' and 'T' form the first entries in the dictionary of phrases. The parsing procedure now proceeds recursively finding each new phrase in the sequence in turn.

The Lempel-Ziv parsing procedure:

1. Search for the longest oligomer subsequence *S *that has appeared in the dictionary.

2. Add *S *concatenated with the next nucleotide in the genome as a new, previously unobserved, phrase into the dictionary.

3. If the sequence is exhausted without discovering a new phrase, exit.

4. Else, with the pointer set to the location following the last observed nucleotide, go back to step 1.

Let *d(n) *denote the number of dictionary items that have been generated after parsing *n *consecutive symbols in the genome string. Then, the number of bits needed to describe the dictionary up to this point provides a simple compression mechanism to describe the entire string up to this point. The pervasive utility of Lempel-Ziv rests upon the observation that such a description is compact and efficient. The theoretical basis for the Lempel-Ziv algorithm is discussed briefly in the Appendix.

All the Java code for implementing the above algorithms and calculating the statistics for the figures and tables is freely available from the authors.

### Statistical methods

All statistical analyses were performed in R. For the analysis of 10-mer sequence space coverage, Figure 2 shows that the association with genome size is non-linear, so we log transformed genome size. In addition, the chance of evenly covering sequence space in a random genome decreases as the frequency of nucleotides is skewed from a uniform distribution. Thus, we transformed GC content by taking the absolute value of (0.5 – GC content). These transformed variables were used as predictors of coverage in a multivariate linear regression that included an interaction term between the predictors. Both variables and their interaction were significant at p < 0.01. We used Cook's distance to identify the top 1% of genomes that have the strongest influence on the regression analysis. We excluded those five genomes and refit the multivariate regression model. We then used that model to predict the coverage values of the five excluded genomes based on their log genome sizes and GC content deviation from 0.5. Only one of the five genomes had a 10-mer coverage value that fell outside the 99.9% prediction range and was therefore identified as an outlier in the model.

## Authors' contributions

CCM conceived the study. ZL performed all the analyses. SSV carried out the theoretical entropy analysis. CCM, ZL and SSV wrote the paper. All authors approved the final manuscript.

## Appendix

### Theoretical basis for the Lempel-Ziv algorithm

The Lempel-Ziv procedure forms a very competitive and computationally appealing entropy estimator for long, complex sequences like the human genome. The technical justification for the Lempel-Ziv algorithm in such contexts is provided by a fundamental convergence theorem: *the superior limit of the ratio of the number of bits needed to describe the Lempel-Ziv dictionary of phrases to the length of the sequence almost surely bounds the entropy rate of any ergodic, stationary random sequence from below *[50]. More formally, if the entropy rate of the source *X *is denoted by *H*(*X*), then

$$\underset{n\to \infty }{\lim\sup}\frac{d(n)\log_{2}(d(n))}{n}\leq H(X)$$

Theorems of this nature form at once the motivation and the ultimate justification of the procedure. The estimates obtained converge very quickly as seen in Figure 5.

### Theoretical analysis of the random pseudo-human genome

Clustering and repetitions of *n*-mers in the human genome over and beyond the chance fluctuations that govern a truly random sequence will manifest themselves in a lower coverage of sequences in *n*-mer space than can be accounted for by chance. It is informative to consider the *n*-mer coverage of a random pseudo-human genome of the same length as the human genome and with the nucleotides A, C, G, and T appearing with equal frequency in the genome. This model is equivalent to a sequence of dinucleotides chosen by independent sampling from the set of sixteen dinucleotides {AA, AC, ..., TG, TT}, each dinucleotide selected with equal probability. This permits a more refined comparison with the human genome in view of features such as the marked depletion of CG dinucleotides in mammalian genomes [51], which are not captured by single nucleotide frequencies alone. As we shall see, however, from an analytical perspective, a consideration of dinucleotide frequencies makes very little difference to the conclusions. The *n*-mer space coverage of the pseudo-human genome will bound from above the corresponding *n*-mer space coverage of the human genome (see Figure 1), the discrepancy between the two providing evidence of statistical clustering or bunching of *n*-mers in the human genome. Such discrepancies in coverage are manifested in the entropy rate: the pseudo-human genome has entropy rate equal to the maximal value log_2 _4 or 2 bits per symbol and bounds from above the entropy rate of the human genome (Figure 5).

Accordingly, consider the simplest model of a random pseudo-human genome as a sequence of nucleotides drawn by independent sampling from the alphabet {A, C, G, T}, with each nucleotide having equal probability of selection. The *4*^*n *^possible *n*-mers each appear with equal probability in the nucleotide sequence. If one considers a random sequence of *t n*-mers (corresponding to a ''genome'' length of *N *= *nt *base pairs), the number of *n*-mers that are absent in the sequence follows the classical coupon collector's paradigm [52]. The probability that exactly *m *of the possible *n*-mers is absent in the random sequence is given by

$$P_{m}=\sum_{k=0}^{4^{n}-m}{\left(-1\right)}^{k}\left(\begin{array}{c} m+k\\ k \end{array}\right)\left(\begin{array}{c} 4^{n}\\ m+k \end{array}\right){\left(1-\frac{m+k}{4^{n}}\right)}^{t}.$$

When *t *is large, say of the order of one billion as in the human genome, the probabilities *P*_*m *_are approximated by a Poisson distribution. More precisely, if

$$n=\frac{1}{\log4}\left[\log(t)-\log\log(t)+\frac{\log\log(t)}{\log(t)}-\frac{c}{\log(t)}+O\left(\frac{\log\log{\left(t\right)}^{2}}{\log{\left(t\right)}^{2}}\right)\right]$$

where logarithms are to the Napier base *e*, *c *is any fixed real constant, and the order term represents a quantity that grows asymptotically no faster than the vanishing term log log(*t*)^2^/log(*t*)^2 ^as *t *becomes large, then the number of excluded *n*-mers approaches a Poisson distribution with mean exp(-*c*) for large values of *t*. More specifically, the probability that exactly *m n*-mers are excluded in the random sequence is asymptotically given by

$$P_{m}\to e^{-e^{-c}}\left(\frac{e^{-mc}}{m!}\right)$$

for large values of *t*. In particular, the probability that all the *n*-mers are present in the sequence is approximately given by

$$P_{0}\approx e^{-e^{-c}}$$

In view of the very rapid extinction of the double exponential for very small values of *c*, say, between -3 and +3 for typical genome sizes, a small positive *c *will result in a probability close to one that all *n*-mers are present while a small negative *c *will result in gaps in coverage with overwhelming probability. As the term involving *c *is sub-dominant in the expression for *n *in terms of *t*, a *phase transition *in *n*-mer coverage in the random pseudo-human genome occurs around the critical value of

$$n\approx \frac{1}{\log(4)}\left[\log(t)-\log\log(t)\right]$$

For instance, when *t *is one billion, of the order of the size of the human genome, the critical value for *n *is 13: with overwhelming probability all n-mers of length 13 or fewer will be found in the random pseudo-human genome with uniform nucleotide frequencies, while there is a breakdown in coverage for *n*-mers of length in excess of 14.

The results are not materially affected if the uniform nucleotide frequencies in the random pseudo-human genome are replaced by the actual observed nucleotide frequencies of 0.295, 0.204, 0.205, and 0.296 for the nucleotides A, C, G, and T in the human genome, or even, in a slightly more refined calculation, the pseudo-human genome is constructed by independent sampling from the set of sixteen dinucleotides with each dinucleotide appearing not with equal probability but in the same frequency as in the human genome (the CG dinucleotide, in particular, being markedly depleted [51]). The *n*-mer coverage for the actual distribution of nucleotide (or dinucleotide) frequencies is bounded below by the coverage of *n*-mer space by a uniform random sequence over an alphabet of size 5 as the probability 1/5 = 0.2 of selecting a given nucleotide (or probability 1/25 = 0.04 of selecting a given dinucleotide) from an alphabet of size 5 lies below the observed nucleotide (respectively, dinucleotide) frequencies in the human genome. We can get increasingly conservative bounds in this fashion by increasing the alphabet size as, under uniform selection, the nucleotide frequency decreases inversely proportional to alphabet size, while the dinucleotide frequency decreases inversely with the square of the alphabet size. The size of alphabet, however, makes little difference to the point where a phase transition in *n*-mer coverage is observed as the expression for the phase transition point for *n *is relatively insensitive to alphabet size (the expression for *n *depends only on the logarithm of the alphabet size). Consequently, random pseudo-human genomes with nucleotide and dinucleotide frequencies matching that of the human genome exhibit an essentially complete coverage of *n*-mers with *n *up to 13, with gaps in coverage occurring abruptly for *n*-mers of size 14 and beyond. For the analysis in Figure 1, we generated a random pseudo-human genome with the same dinucleotide frequencies as the human genome by using a first-order Markov process with transition probabilities that match the probabilities for each nucleotide following the previous nucleotide in the human genome. The substantial agreement of the stochastic simulation results for the pseudo-human genome in Figure 1 with the theoretical predictions serves to provide a validation of the sampling methodology.

## Supplementary Material

Additional file 1 **Supplementary-1.** Space coverage of n-mer space (1–20) for *Homo sapiens, Mus musculus, Drosophila melanogaster, Caenorhabditis elegans, Arabidopsis thaliana, Saccharomyces cerevisiae*, and *Escherichia coli *(K-12) genomes by the stochastic searching algorithm. Click here for file

Additional file 2 **Supplementary-2.** The 10-mer space coverage for 433 fully sequenced microbial with genomes GC content and genome sizes. Click here for file

Additional file 3 **A full list of high frequency *n*-mers for *Homo sapiens *genome.** Click here for file

Additional file 4 **Supplementary-4.** The NCBI genome sequence accession ID for *Drosophila melanogaster*, *Arabidopsis thaliana*, *Caenorhabditis elegans*, *Saccharomyces cerevisiae*. Click here for file

Additional file 5 **Supplementary-5.** The NCBI genome sequence accession ID for 433 microbial. Click here for file
